# The Safety, Feasibility, and Efficacy of a Structured Individual Exercise Program for Community-Dwelling Stroke Patients

**DOI:** 10.3390/healthcare12222281

**Published:** 2024-11-15

**Authors:** Jung-Lim Lee, Sung-Hwa Ko, Sungchul Huh, Jong-Chul Jung, Soo-Yong Kim, Dong-Yun Bae, Yong-Il Shin, Yuna Kim

**Affiliations:** 1 Research Institute for Convergence of Biomedical Science and Technology, Pusan National University Yangsan Hospital, Yangsan 50612, Republic of Korea; lim6668@naver.com (J.-L.L.); dr.huhsc@gmail.com (S.H.); kendoh98@hanmail.net (J.-C.J.); gasigogi11@naver.com (S.-Y.K.); way1813@naver.com (D.-Y.B.); rmshin01@gmail.com (Y.-I.S.); kyoona1289@gmail.com (Y.K.); 2 Department of Rehabilitation Medicine, Pusan National University Yangsan Hospital, Yangsan 50612, Republic of Korea; 3 Department of Rehabilitation Medicine, Pusan National University School of Medicine, Busan 50612, Republic of Korea; 4 Department of Physical Therapy, Pusan National University Yangsan Hospital, Yangsan 50612, Republic of Korea

**Keywords:** stroke, comprehensive exercise program, structured individual exercise, safety, feasibility, efficacy

## Abstract

Introduction: Stroke is a leading cause of mortality and long-term disability worldwide, often resulting in low levels of physical activity post-discharge. A comprehensive exercise program could be a safe method to increase stroke patients’ physical activity and exercise volume. This pilot study examines the effectiveness, safety, and feasibility of a comprehensive exercise regimen for stroke patients residing in the community after discharge. Methods: This single-group pilot study included patients who received inpatient rehabilitation for motor impairment following a stroke. Community-dwelling participants engaged in a comprehensive exercise program, which was structured and individualized, consisting of stretching, aerobic exercise, strength training, and balance exercises, conducted under the supervision of a health exercise specialist in a dedicated living lab within the hospital. The program involved 20 sessions, each lasting 50 min, conducted three to five times weekly. The pre- and post-intervention evaluations were comprehensive, including safety and validity assessments, along with physical function tests such as cardiopulmonary exercise testing and various fitness tests (6 min walk test, timed up and go test, chair stand test, figure-of-8 walk test, grip strength test, and sit-and-reach test). Depression levels were assessed using the Geriatric Depression Scale (GDS). Results: A total of 22 subjects were recruited, with 21 completing the study after one dropout due to fatigue and visiting distance. Among the 21 completers, one subject experienced a mild adverse event (hypoglycemia), which was not serious, confirming the program’s safety. The exercise participation rate was 95.5%, and the adherence rate averaged 95.2%, with all completers achieving over 80% adherence. Significant improvements were observed in VO_2_ peak (*p* = 0.000), 6 min walk test (*p* = 0.000), timed up and go test (*p* = 0.000), chair stand test (*p* = 0.000), figure-of-8 walk test (*p* = 0.000), and grip strength (*p* = 0.001). However, changes in the peak respiratory exchange ratio (*p* = 0.078), sit-and-reach test (*p* = 0.228) and GDS scores (*p* = 0.275) were not significant. Conclusion: This study demonstrates that the comprehensive exercise program is sufficiently safe and valid for stroke rehabilitation patients. The program significantly enhances cardiopulmonary endurance, walking ability, balance, muscle strength, muscle endurance, and coordination. Further research with larger sample sizes and control groups is needed to confirm these findings and explore additional benefits.

## 1. Introduction

Stroke remains a leading cause of long-term disability worldwide, significantly impacting the quality of life of survivors [1]. It is of the utmost importance that patients with stroke engage in regular exercise, particularly during the transition from hospital-based care to independent community living. Effective exercise programs are essential to facilitate this transition to improve functional outcomes, reduce the risk of secondary complications, and enhance overall quality of life [2]. Most exercise programs are conducted in hospitals; however, interest in community-based group programs for physical activity and exercise is increasing [3]. This shift in focus from hospital to community settings is crucial, given the growing prevalence of stroke and the increasing need for sustainable long-term rehabilitation solutions.

A 2020 Cochrane review of 75 studies and 3617 stroke patients analyzed the effects of aerobic, strength, and comprehensive exercises and their effect on functional improvement after stroke [2]. These findings indicated that aerobic exercise is associated with improved disability, cardiorespiratory function, balance, and walking ability. Strength training is associated with enhanced strength, walking ability, and balance. Combined aerobic and resistance training is associated with improved disability, walking ability, and balance. The evidence for the efficacy of aerobic and strength training was intense, whereas evidence for comprehensive exercise was weak, owing to the small number of studies.

Despite the weaker evidence for comprehensive exercise, the American Stroke Association’s statement of physical activity and exercise recommendations for stroke survivors states that physical activity and exercise after stroke are strongly recommended for comprehensive exercises, including aerobic exercise as well as strength and flexibility exercises. Furthermore, the guidelines highlight the importance of prescribing physical activity and exercise tailored to an individual’s physical condition following a stroke. This approach allows for a more personalized and effective exercise program than a one-size-fits-all approach [4].

However, numerous studies investigating the actual physical activity state of stroke survivors have shown that it is very low [5,6,7,8]. Even the exercise levels suggested in the guidelines show a large gap. This gap is due to several barriers. A systematic review reported published studies on perceived barriers to and motivators for physical activity after stroke, and the results showed that the most reported barriers were lack of motivation, environmental factors (e.g., transport), health concerns, and stroke impairment [9]. Additionally, a recent survey also highlighted barriers such as physical limitations due to disabilities, lack of facilities, and health concerns. As a means of overcoming these barriers, community-dwelling stroke patients expressed a desire for an accurate assessment of their condition and individualized exercise programs [10]. Therefore, a comprehensive exercise program that stroke patients can perform in the community after discharge is needed, and this should be both structured and individualized according to the physical activity guidelines for stroke patients. The aims of our study were (1) to evaluate the safety and feasibility of a structured exercise program for community-dwelling stroke patients, ensuring that it can be safely administered without adverse effects, and (2) to assess the program’s efficacy in improving various physical functions.

## 2. Materials and Methods

### 2.1. Study Design and Setting

This was a single-arm study involving patients with stroke. Participants underwent a comprehensive exercise program and pre-intervention assessments. The intervention was conducted by a health exercise manager (The professional field in Korea qualified to provide physical education to patients, consisting of those who majored in physical education, holding nationally recognized certifications) and consisted of 20 sessions (3–5 times per week for 4–7 weeks). The study was conducted from 19 May 2022 to 30 September 2023 at Pusan National University Yangsan Hospital in Yangsan, Gyeongsangnam-do. This study was approved by the Institutional Review Board of Pusan National University Yangsan Hospital (IRB No. 04-2022-016), and written consent was obtained from all participants or their representatives. This clinical trial was registered in the Clinical Trials Registry (ID: NCT05616858 Complex Exercise Program for Stroke Patients).

### 2.2. Participants

Inclusion criteria were: (1) stroke patients aged 40 or older, discharged from the rehabilitation medicine department at Pusan National University Yangsan Hospital, and residing or planning to reside in the community; (2) capable of independent walking with a Functional Ambulation Category (FAC) score of four or higher; (3) minimal dependency in daily living activities with a Modified Rankin Scale (mRS) score of two or less; (4) minimal or no spasticity in the affected upper and lower limbs with a Modified Ashworth Scale (MAS) score of one or less; and (5) a Manual Muscle Test (MMT) score of three (F grade) or higher for the three major joints of the affected upper and lower limbs. The exclusion criteria were as follows: (1) moderate to severe cognitive impairment with a Korean Mini-Mental State Examination (K-MMSE) score below 18; (2) moderate or higher fall risk with a Berg Balance Scale (BBS) score below 41; (3) severe language impairment hindering the understanding or expression of symptoms related to the exercise program; (4) severe cardiovascular conditions making participation in the exercise program unfeasible; (5) musculoskeletal conditions preventing exercise (e.g., degenerative arthritis, rheumatoid arthritis, sprains, fractures, and other conditions that limit joint mobility); and (6) other conditions deemed inappropriate by a rehabilitation medicine specialist.

### 2.3. Intervention

The intervention program consisted of 20 sessions, provided at least 3–5 times per week, and included a 50 min comprehensive exercise program. The program was structured as follows: 5 min of warm-up (flexibility exercises), 40 min of main exercise (20 min of aerobic exercise and 20 min of strength and balance exercises), and 5 min of cool-down (flexibility exercises). The program was developed with physical activity and exercise recommendations for stroke survivors by the American Stroke Association [4]. The intervention was conducted one-on-one by an exercise specialist in a dedicated living laboratory space separate from the physical therapy area, where participants could receive the intervention. Before starting the exercise program, the participants underwent a medical examination to confirm their suitability for a comprehensive exercise program.

#### 2.3.1. Warm-Up and Cool-Down

The flexibility exercises included in the warm-up and cool-down sessions were designed to relax the neck, triceps brachii, biceps brachii, anterior arm, lower back, quadriceps, biceps femoris, ankle joints, and muscles. Each movement involved holding the static stretches for 10–30 s.

#### 2.3.2. Main Exercise

The main exercise program was prescribed based on the participants’ cardiorespiratory fitness and 1RM (one-repetition maximum) strength assessed before the intervention. The exercise regimen was divided into two types, A and B, alternating between sessions. The types were designed with a focus on optimizing the efficiency of strength training: Type A targeted anterior muscle groups, while Type B concentrated on posterior muscle groups. To keep participants motivated and avoid monotony, different exercises were also applied for aerobic and balance training according to each type. Strength training was started at 50% of the assessed 1RM during the first week and progressed through four stages by increasing the intensity by 10% every five sessions. If the participants did not achieve an 80% completion rate for the three sets of 10 repetitions, they remained at the same weight and performed an additional 5 repetitions. For balance exercises, the difficulty was adjusted to maintain a Rating of Perceived Exertion (RPE) of 11–14 using tools such as mats and small equipment, with participants completing three sets of either 10 repetitions or 10 s each.

- A type

Aerobic exercises were conducted in an interval training format using a stepper in a seated position for the A type. The intensity was alternated every 2 min, with high intensity set at 60% heart rate reserve (HRR) and a rating of perceived exertion (RPE) of 11–14 (somewhat hard) and low intensity set at 30% HRR and an RPE of 9–11 (light). For strength and balance exercises, shoulder press, chest press, biceps curl, hip flexion, and knee extension exercises were used for strength training. In contrast, tandem exercises and sit-up exercises were used for balance training.

- B type

For type B, aerobic exercise was conducted using a treadmill in a standing position, following the same interval training format as for type A. Strength exercises included seated back rows, triceps extension, hip abduction, and knee flexion. Balance exercises included squats, one-leg standing, and bridge exercises, which provided a comprehensive full-body workout.

### 2.4. Outcomes

#### 2.4.1. Safety

Participants were asked to record the details of all adverse events (AEs) and the specifics of all completed exercises (e.g., type, intensity, and duration). Safety data were monitored and evaluated using adverse event reports, which defined AEs as unexpected medical occurrences that may or may not have a causal relationship with the intervention (exercise).

#### 2.4.2. Feasibility

Feasibility was assessed by combining information from training logs and records maintained by physical therapists to evaluate adherence to the intervention. The participants were considered adherent if they attended at least 80% of the sessions (16–20 sessions) and completed 80% or more of the prescribed exercises. The 1RM (one-repetition maximum) and Borg 6–20 RPE (rating of perceived exertion) scales were used to report the intensity level of physical activity and exercise.

#### 2.4.3. Physical Functions

All participants underwent an assessment of their exercise function before and after the intervention. The evaluation included cardiopulmonary endurance assessed using the cardiopulmonary exercise testing (*CPET*), cardiorespiratory and walking function tested with the 6 min walk test (6MWT), balance tested with the timed up and go (TUG), strength assessed with grip strength, muscular endurance evaluated with the 30 s chair stand test, coordination assessed with the figure-of-8 walk test, and flexibility evaluated with the sit-and-reach test. All physical function assessment tools, except the CPET, are used in South Korea as part of the national policy for measuring physical fitness in older adults, known as the “National Fitness 100”.

- CPET

In this study, CPET was used to assess participants’ cardiopulmonary capacity and examine the response of the heart and lungs. The CPET was conducted on a treadmill using METALYZER 3 B equipment (CORTEX INC., Leipzig, Germany). The parameters analyzed included VO_2_ peak and peak respiratory exchange ratio (RER). The reliability of variables during CPET in patients with stroke was high (intraclass correlation coefficient [ICC] = 0.93) [11].

- 6MWT

For the 6MWT, a rectangular track measuring 20 m × 5 m, totaling 50 m, was set up with cones placed at each corner. The participants were instructed to walk around the track as quickly as possible for 6 min, ensuring their feet did not leave the ground, thus avoiding running. At the end of the test, the location where the participant stopped was marked, and the total distance walked (in meters) was measured. This assessment tool demonstrates high test-retest reliability in individuals with stroke, with a weighted mean of raw correlation coefficients of 0.98 (95% confidence interval [CI] = 0.98 0.99) [12].

- TUG

The participants were seated in the center of a chair approximately 40 cm high, with their hands resting on their thighs. At the start of the signal, the participant stood up and walked as quickly as possible to a cone placed 3 m away, circled around it, and returned to sit back on the chair. The time required to complete each task was recorded. This assessment tool has demonstrated excellent intra-rater reliability (ICC = 0.99) and test-retest reliability (ICC range = 0.97–0.98) in community-dwelling individuals aged 60 and older and also showed excellent test-retest reliability (ICC range = 0.935–0.999) in community-dwelling adults aged 50 and older [13].

- Chair stand test

The participants sat in the center of a chair approximately 40 cm high with their arms crossed in front of their chest. At the start signal, the subjects were instructed to stand up and sit down repeatedly for 30 s. The total number of times they could stand fully during the 30 s was recorded. High agreement was also demonstrated for this assessment, with ICC values ranging from 0.87 to 0.94 [14].

- Figure-of-8 Walk Test

A rectangular area measuring 3.6 m × 1.6 m was marked on the floor, and cones were placed at the corners. A chair was positioned 2.4 m from each cone. The participants began the test while seated in a chair positioned at the center of the rectangle. At the start signal, the participants walked around the cone positioned at the right rear, returned to the chair, sat down, immediately walked around the cone positioned at the left rear, and returned to the chair. This sequence was repeated twice, and the total time taken was recorded. This assessment also showed excellent intra-, inter-, and test-retest reliability in individuals with stroke, with ICC values ranging from 0.944 to 0.999 [15].

- Grip strength

The participant stood with feet shoulder-width apart and grasped the dynamometer’s handle with the second knuckle of the fingers, keeping the arm straight and maintaining a 15° angle between the arm and the torso. At the start of the signal, the participant squeezed the dynamometer with maximum force for 5 s. The test was performed twice for each hand, and the highest value was recorded. The average of the maximum values obtained from both hands was used as the final result. A Jamar smart hand dynamometer (Performance Health International Ltd., UK) was used for measurements. This assessment is reliable in individuals with stroke, with ICCs ranging from 0.97 to 0.99 [16].

- Sit-and-Reach Test

The participants sat on the floor with their legs extended and feet flat against the measurement device. With their arms extended forward, the participants were instructed to reach forward as far as possible by pushing the measuring plate with their fingertips. The test was performed twice, and the maximum distance was recorded. A sit-and-reach test box (Shinahsports, Republic of Korea) was used for the measurements. The within-session reliability of this assessment was found to be good, with an ICC of 0.98, and the between-session reliability was also rated as good, with an ICC of 0.79 [17].

#### 2.4.4. Mood

The Geriatric Depression Scale (GDS) was used to assess changes in depression levels before and after participation in the comprehensive exercise program and mood changes in participants following the program. According to a 2009 study, the GDS is an assessment tool for detecting mild depression in patients with stroke. Its test-retest reliability analysis showed a high Pearson’s correlation coefficient (r = 0.75), indicating high reliability. Originally developed as a depression scale for older adults, this assessment tool is also used for diagnosing depression in stroke patients, including younger individuals, due to its simplicity and adaptability [18].

### 2.5. Statistical Analyses

Data analysis was performed using SPSS (Statistical Package for the Social Sciences) version 23.0 (SPSS INC., Chicago, IL, USA). The demographic data were analyzed using the descriptive statistics. A paired t-test was used to compare the pre- and post-intervention data. The results were considered statistically significant if the *p*-value was less than 0.05.

## 3. Results

### 3.1. General Charateristics

**Figure 1:**
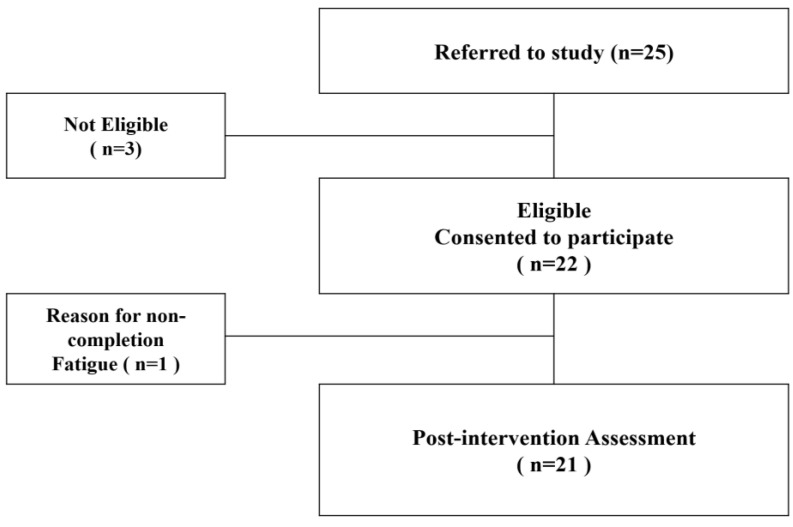
Flow of participants during the trial.

A total of 25 post-stroke patients applied for recruitment in the study. Of these, two patients were excluded as their MMT scores for major joints did not meet the inclusion criteria, and one patient was excluded for not meeting the MAS inclusion score (Figure 1). Therefore, 22 participants took part in the intervention. Their demographic characteristics are shown in Table 1. The participants included 22 participants, with an average age of 59.48 ± 11.25 years and an average height of 166.30 ± 7.88 cm. The average weight was 66.73 ± 9.77 kg, and the average time since the onset of stroke was 28.05 ± 18.46 months. The sex distribution was 68.18% male and 31.82% female. Stroke types were ischemic (72.73%) and hemorrhagic (27.27%). Regarding the affected side, 27.27% had left-sided hemiparesis, and 72.73% had right-sided hemiparesis. A total of 40.91% had hypertension, and 27.27% had diabetes as a pre-existing condition.

### 3.2. Safety

In the adverse event report used to determine the occurrence of adverse events, one of the 21 participants experienced a mild adverse event (hypoglycemia), which was possibly related to exercise. However, this was not classified as a serious adverse event. The affected participant was instructed to discontinue exercise on that day, undergo medical evaluation, and rest.

### 3.3. Feasibility

**Figure 2:**
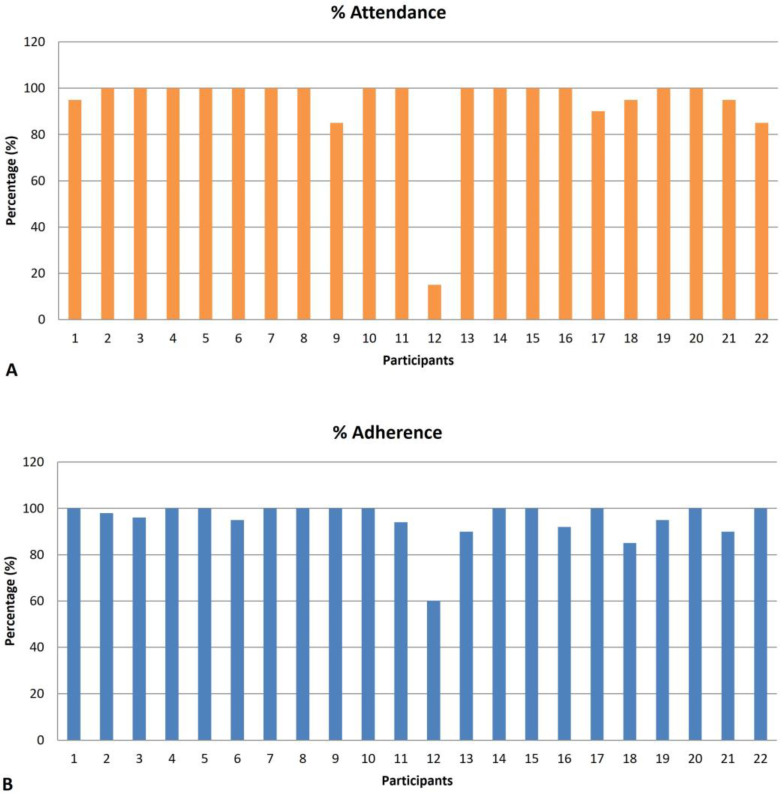
Feasibility of comprehensive exercise program in participants. (**A**) Attendance. (**B**) Adherence.

Of the 22 participants in the comprehensive exercise program, 21 attended at least 80% of the 20 intervention sessions, resulting in a participation rate of 95.5%. One participant withdrew after attending three sessions (6%) because of fatigue and travel distance. The exercise compliance rate was also high, with an average completion rate of 95.2%, indicating that all remaining participants completed at least 80% of their assigned exercise sessions (Figure 2).

### 3.4. Effects on Physical Functions

To further examine the effects of the interventions, Cohen’s *d*-values were calculated using the formula *d = M*_1_*−M*_2_*/SD_pooledd_*, where *M*_1_ and *M*_2_ denote the pre- and post-intervention means for the participants, respectively, and *SD_pooledd_* is the pooled standard deviation derived from these scores. This calculation allows for an assessment of the magnitude of the difference between groups, offering insight into the practical significance of the intervention effects, independent of sample size.

In CPET, the VO_2_ peak significantly increased from 25.14 ± 5.42 mL/kg/min to 27.62 ± 5.91 mL/kg/min (*p* < 0.001), with a moderate effect size (Cohen’s *d* = 0.44). The peak RER also increased from 1.15 ± 0.11 to 1.18 ± 0.11, but this change was not statistically significant (*p* = 0.078) and demonstrated a small effect size (Cohen’s *d* = 0.27).

6MWT distance increased significantly from 415.74 ± 117.90 m to 508.64 ± 123.93 m (*p* < 0.001), with a moderate-to-large effect size (Cohen’s *d* = 0.77). The timed up and go (TUG) test completion time decreased significantly from 11.27 ± 4.75 s to 8.27 ± 3.46 s (*p* < 0.001), with a moderate to large effect size (Cohen’s *d* = −0.72). The chair stand test showed a significant increase in repetitions from 17.48 ± 4.50 to 22.14 ± 6.43 (*p* < 0.001), with a large effect size (Cohen’s *d* = 0.84). The figure-of-8 walk test completion time decreased significantly from 43.58 ± 16.47 s to 32.72 ± 15.04 s (*p* < 0.001), with a moderate to large effect size (Cohen’s *d* = −0.69). Grip strength also increased significantly from 29.06 ± 6.88 kg to 34.34 ± 7.73 kg (*p* = 0.001), with a moderate to large effect size (Cohen’s *d* = 0.72). In contrast, the sit-and-reach test score showed a slight, non-significant increase from 3.80 ± 9.71 to 5.81 ± 8.68 (*p* = 0.228), with a small effect size (Cohen’s *d* = 0.22) (Table 2).

### 3.5. Mood

The change in depression levels measured by the GDS decreased from 12.57 ± 8.91 points to 11.48 ± 9.24 points, though this reduction was not statistically significant (*p* = 0.275), with a very small effect size (Cohen’s d = −0.12) (Table 2).

## 4. Discussion

We found that the structured individual comprehensive exercise program was safe and feasible for community-dwelling patients with stroke. Additionally, we confirmed that it effectively improved cardiovascular endurance, walking ability, balance, muscle strength, muscular endurance, and coordination.

According to a previous study [10], stroke patients in the community face various social and personal barriers, such as disabilities, lack of appropriate facilities, concerns about their health conditions, and limited accessibility. In particular, 29% of the community patients with stroke who participated in the survey reported that their disability was a barrier, while 13% cited concerns about their health. This highlights the need for appropriate methods and exercise programs that patients with stroke and disabilities can safely participate in. Therefore, this study aimed to develop and implement a comprehensive exercise program that allows community stroke patients to participate without concerns regarding their disabilities or health and to verify its safety and effectiveness.

Numerous studies have demonstrated the effectiveness of exercise in stroke patients [2,19,20]. A Cochrane review published in 2020 analyzed 75 studies involving 3617 stroke patients to assess the impact of various physical activities and exercises, including aerobic, resistance, and mixed exercises, on post-stroke functional recovery. A previous study reported that aerobic exercise significantly improves disability levels, cardiovascular fitness, balance, and walking ability, with relatively strong evidence supporting these findings [2]. Additionally, high-intensity resistance training has significantly increased muscle strength and improved functional independence and quality of life in patients [19]. Combined exercise programs that integrate the benefits of multiple exercise types have also proven effective. A 2006 study demonstrated that a combined program that included task-oriented resistance training and aerobic exercise led to significant improvements in muscle strength and functional performance in patients with stroke [20]. We developed a comprehensive exercise program that included machine-based adjustable resistance training, structured aerobic exercises, balance training, and flexibility exercises. The program was administered to 22 community-dwelling patients with stroke to verify its validity and safety.

We developed a personalized, comprehensive exercise program for community-dwelling patients with stroke by referencing the existing exercise program guidelines for stroke patients in Korea and the criteria outlined in a study by the American Heart Association and American Stroke Association [4]. Prior to participating in the program, patients were required to undergo an evaluation by a specialist to determine their suitability for the exercise regimen. Additionally, individual exercise capacity (such as 1RM and RPE measurements) was assessed before starting the program. The exercise intensity was designed to increase by 10% weekly (five sessions per week) based on the individual’s performance level.

No serious adverse events occurred among the 22 participants who completed 21 post-stroke community-based comprehensive exercise program sessions. One minor adverse event (hypoglycemia) was associated with an underlying condition (diabetes) but was not a severe reaction. The remaining participants completed the program safely. The presence of an exercise specialist who provided one-on-one guidance throughout the program ensured that no major adverse events occurred, allowing all participants to finish the study safely and confirming the program’s safety.

The feasibility of the post-stroke community-based comprehensive exercise program was demonstrated by a participation rate of 95.5%, with the participants attending more than 80% of the sessions. The program also showed a high adherence rate (95.2%), which was comparable with the average compliance rates observed in 11 other studies on combined exercise programs [21].

To evaluate the effects of the comprehensive exercise program, we assessed cardiovascular fitness, gait ability, balance, muscle strength, coordination, and flexibility. Cardiopulmonary exercise testing (CPET) was used to measure cardiovascular fitness. Among the CPET results, VO_2_ peak and peak RER were chosen as the outcome measures. VO_2_ peak is widely recognized in many studies as a critical indicator of maximum cardiopulmonary function during exercise [22]. In our study, VO_2_ peak showed a significant improvement (*p* < 0.001), indicating that the comprehensive exercise program contributed to enhanced cardiopulmonary function, increasing maximum oxygen consumption. Peak RER refers to the highest respiratory exchange ratio, defined as the ratio of VCO_2_ to VO_2_. Typically, RER values range from 0.7 to 0.8 during low-intensity exercise, 1.0 at moderate intensity, and 1.1 to 1.3 at high-intensity exercise [23]. Our study’s peak RER increased from 1.15 ± 0.11 to 1.18 ± 0.11, but the change was not statistically significant (*p* = 0.078). This is likely because the participants were already at a relatively high exercise intensity level before the program, resulting in a smaller change in the peak RER.

In the results of this study, 6MWT, which measures both cardiopulmonary fitness and gait ability, showed a statistically significant improvement (*p* < 0.001). The 6MWT is primarily used to evaluate the effects of aerobic exercise and gait training [24]. The substantial change in this study’s comprehensive exercise program, despite the short intervention period (4–7 weeks), was likely due to the personalized nature of the program. Similarly, the timed up and go test, which assesses balance, showed statistically significant results (*p* < 0.001). The chair stand test, a reliable measure of muscular endurance in stroke patients [14], and the figure-of-8 walk test, which assesses advanced walking ability in stroke patients [15], also demonstrated significant improvements following the comprehensive exercise program. Regarding grip strength, previous research [25] indicated that upper-extremity exercise programs for community stroke patients did not result in significant changes in grip strength compared with a control group. However, our comprehensive exercise program observed substantial improvement in grip strength. It should be noted that the previous study included patients with varying degrees of disability (mild, moderate, and severe), while our study focused on participants with relatively mild impairments. This suggests that the effect of the exercise program may vary depending on the degree of impairment, which must be considered when interpreting the differing results. Flexibility was assessed using the sit-and-reach test, but it did not significantly affect the program. In terms of depression, as measured by the GDS, there was a trend toward a reduction in depressive symptoms before and after the program, but the change was not statistically significant. This may be because depression is influenced by various factors, making it challenging to detect substantial changes during short-term exercise interventions.

The comprehensive exercise program used as the intervention in this study was more systematic in terms of frequency, intensity, time, and type compared with previous research on combined exercise programs [2,20,26]. Compared with another previous national study [3], which used a new exercise program within the intervention and focused on verifying effectiveness with a control group, this study used a more personalized program with one-on-one expert supervision, emphasizing the demonstration of the program’s safety and validity under individualized professional supervision. It was designed to be carried out using community-based exercise facilities while offering the advantage of a personalized program tailored to adjust exercise intensity according to each patient’s capacity. As stroke patients may face safety issues when using exercise equipment designed for the general population, accurate initial assessment and medical evaluation are essential. To ensure safety, trained exercise professionals, such as physical therapists or sports/exercise specialists for health, educated patients on proper posture and exercise techniques through one-on-one supervision and were prepared to respond to emergencies. As demonstrated by the results, the comprehensive exercise program developed by the research team proved to be both safe and feasible while also showing improvements in specific physical abilities. This pilot study has a limitation that constrains the generalizability of its results, most notably the small sample size and absence of a control group. To validate the intervention’s efficacy more robustly, future research should include larger sample sizes and control groups. To extend this comprehensive exercise program, it will require not only community-based exercise facilities but also education and management for specialized personnel for community-dwelling stroke patients. Additionally, financial support from the government would also be crucial to ensure the effectiveness and quality of the program.

## 5. Conclusions

This study demonstrates that a comprehensive exercise program for community-dwelling patients with stroke is both safe and feasible. The participants showed significant improvements in cardiopulmonary endurance, walking ability, balance, strength, muscular endurance, and coordination. These findings suggest that this program is an effective rehabilitation method for facilitating physical recovery in patients with stroke. However, there were no statistically significant changes in depression levels, indicating the need for additional psychological interventions. Overall, a comprehensive exercise program is a safe and beneficial approach for post-stroke rehabilitation. However, for the successful extension of this study, adequate indoor space, appropriate exercise facilities, and trained professionals capable of organizing and administering the exercise program are essential. Furthermore, national financial support would be crucial to ensure the feasibility and sustainability of such initiatives.

## Figures and Tables

**Table 1:** General characteristics of participants.

Variable	Participants (*n* = 22)
Age (years)	59.48 ± 11.25
Height (cm)	166.30 ± 7.88
Weight (kg)	66.73 ± 9.77
Time post-stroke (mons)	28.05 ± 18.46
Sex	
Men	15 (68.18%)
Women	7 (31.82%)
Stroke type	
Ischemic	16 (72.73%)
Hemorrhagic	6 (27.27%)
Hemiparetic side	
Left	6 (27.27%)
Right	16 (72.73%)
Hypertension	
Yes	9 (40.91%)
No	13 (59.09%)
Diabetes	
Yes	6 (27.27%)
No	16 (72.73%)

Values are the mean ± s.d.

**Table 2:** Effects of the structured individual comprehensive exercise.

Variables	Pre	Post	*p*-Value
VO_2_ peak (mL/kg/min)	25.14 ± 5.42	27.62 ± 5.91	0.000 *
Peak RER	1.15 ± 0.11	1.18 ± 0.11	0.078
6 min walk test (m)	415.74 ± 117.90	508.64 ± 123.93	0.000 *
Timed up and go (s)	11.27 ± 4.75	8.27 ± 3.46	0.000 *
Chair stand test (times)	17.48 ± 4.50	22.14 ± 6.43	0.000 *
Figure-of-8 Walk Test (s)	43.58 ± 16.47	32.72 ± 15.04	0.000 *
Grip strength (kg)	29.06 ± 6.88	34.34 ± 7.73	0.001 *
Sit-and-reach test (cm)	3.80 ± 9.71	5.81 ± 8.68	0.228
GDS	12.57 ± 8.91	11.48 ± 9.24	0.275

Values are the mean ± s.d. * *p* < 0.05.

## Data Availability

The dataset analyzed in this study is not publicly available because of ethical and legal regulations regarding personal data protection.
